# How to Determine Glass Transition Temperature of Polymer Electrolytes from Molecular Dynamics Simulations

**DOI:** 10.1021/acs.jpcb.4c06018

**Published:** 2024-10-21

**Authors:** Harish Gudla, Chao Zhang

**Affiliations:** Department of Chemistry-Ångström Laboratory, Uppsala University, Lägerhyddsvägen 1, BOX 538, 75121 Uppsala, Sweden

Polymer electrolytes have garnered significant attention in recent years in energy storage devices such as lithium-ion batteries as a safer alternative to liquid electrolytes.^1^ Understanding polymer electrolytes’ glass transition temperature (*T*_g_) is essential for optimizing and studying their structural dynamics, mechanical properties, thermal stability, and electrochemical performance. The glass transition temperature marks the (nonequilibrium) transition point at which the polymer transforms from a rigid, glassy state to a more flexible, rubbery state. Polymer electrolytes are composed of a polymer matrix doped with ionic species, and their structural dynamics limit the ionic conductivity that is crucial for electrochemical applications. The *T*_g_ of polymer electrolytes dictates the onset of segmental motion within the polymer chains. This leads to increased chain mobility and facilitates coupled ion transport, as understood by free volume theory.^2^ The differences between coupled and decoupled ion transport can be seen from ionic conductivity’s temperature (*T*) dependence (σ). For decoupled transport, this follows a classical Arrhenius relation, i.e., log σ ∝ 1/*T*, while for the coupled transport, it follows a Vogel–Fulcher–Tammann (VFT) behavior, i.e., log σ ∝ 1/(*T*–*T*_0_) where *T*_0_ is the Vogel temperature.^3^*T*_0_ is experimentally found ca. 50 K below the glass transition temperature. The conduction of ions in polymer electrolytes is commonly coupled to the segmental motion of polymer chains and it is generally accepted that a low *T*_g_ is necessary for polymer electrolytes with high conductivity. Therefore, determining the *T*_g_ provides critical insights into the temperature range over which polymer electrolytes can maintain their structural integrity and functionality, guiding the selection of appropriate operating conditions.

Molecular Dynamics (MD) simulations have emerged as indispensable tools for probing the molecular interactions of polymer electrolytes. They provide detailed insights into their dynamic behavior and help understand their complex structure–property relationships. One central aspect to MD simulations is the choice of force field, which defines the mathematical form and parameters governing intermolecular interactions. The accuracy of force fields is crucial for faithfully reproducing polymer electrolytes’ thermal, structural, and dynamical properties. However, force field parameters are often optimized to a set of desired properties and available experimental results. The computed values of *T*_g_ in polymer systems derived from MD simulations tend to exhibit an overestimation averaging between 80 and 120 K when compared to experimental measurements.^4,5^ This disparity can be attributed to limitations in force field parameters or choices in cooling rates,^6^ with MD simulations operating on a significantly faster time scale (10^9^ K/s) compared to experiments (10^–2^–10^–1^ K/s). Thus, utilizing scaled or normalized temperatures with respect to *T*_g_, i.e., *T* – *T*_g_, in MD simulations is recommended when comparing the results with experiments.^7−9^

Therefore, this note aims to discuss and clarify the practical aspects of using MD simulations to calculate the glass transition temperature (*T*_g_). Here we delve into the intricacies of setting up MD simulations, choosing the two different methods of annealing simulations, and analyzing the results in order to extract (*T*_g_). By surveying the literature and assessing computational protocols, we show how one can determine (*T*_g_) of the polymer electrolyte using MD simulations by taking the PEO/LiTFSI (poly(ethylene oxide)/lithium bis(trifluoromethane)sulfonimide) system as an example.

While the simulation protocol for equilibrating the polymer system appears consistent across different studies, various annealing methods have been employed in the MD simulations to obtain *T*_g_. Wu^10^ implemented a stepwise cooling approach, where multiple NPT (constant particle number, pressure, and temperature) simulations were conducted from a higher to lower temperature range, allowing equilibration at each step. The study investigated different properties such as density, specific volume, mean square displacements of polymer systems, and various energy components to determine *T*_g_, revealing that density profiles offer consistent *T*_g_ values for bulk or amorphous polymer systems. On the other hand, Klajmon et al.^11^ utilized a continuous cooling method for annealing simulations of the polyethylene glycol (PEG) system, involving a single NPT simulation at a specific cooling rate. They observed a difference of 10–20 K in the effect of slow (5 K/ns) and fast (40 K/ns) cooling rates on *T*_g_. Also, they found that a hyperbolic fit reduces computational uncertainty compared to the conventional bilinear fit of density profiles. The list of reported *T*_g_ based on MD simulations for the model PEO system is presented in Table 1. Besides the force field-dependence and different annealing methods mentioned already, other factors also contribute to the dispersion in the reported *T*_g_. It is known that the introduction of LiTFSI into the polymer system resulted in a 20–30 K increase in *T*_g_(12) due to the constraining effect on polymer segmental motion caused by the inclusion of salt, which forms physical cross-links between polymer chains. Further, the *T*_g_ shows inversely depend on the polymer molecular weight as described by the Flory–Fox relation,^13^ although MD simulations from Habasaki^14^ and Klajmon et al. do not show a clear trend in *T*_g_ for systems with molecular weights higher than 1 kg/mol.

**Table 1:** Details of the References, Polymer Systems, Force Fields, and Computed *T*_g_

Reference	System	Force Field	Simulated *T*_g_ [K]	Mol. wt.[kg/mol]
Wu^10^	PEO	OPLS	270 (bulk)	2.2
			260 (film)	
Habasaki^14^	PEO	OPLS	250	1, 2
Klajmon et al.^11^	PEG	OPLS	269 ± 21	1–10
Gudla et al.^12^	PEO-LiTFSI	GAFF	285–320	1.1
Webb et al.^15^	PEO-LiTFSI	TraPPE-UA	275	2.5
Fang et al.^16^	PEO-LiTFSI	OPLS-AA	250–260	2.6
Gullbrekken et al.^17^	PEO-LiTFSI	OPLS-AA	250–260	1, 4.4

In the following, we present a MD simulation protocol for polymer electrolyte systems (GroPoB, https://github.com/Teoroo-CMC/GroPoB) and investigate the impact of annealing methods on the determination of *T*_g_. Section 1 of the Supporting Information provides a step-by-step guide in building initial configurations and input files for simulating polymer electrolytes systems. The flowchart of computational protocol, from constructing the polymer electrolyte simulation box to executing the MD simulations, is depicted in Figure 1. The MD protocol for equilibrating the initial configurations commenced with an energy minimization step utilizing the steepest descent algorithm. This was followed by an NVT (constant number, volume, and temperature) ensemble at 400 K for 10 ns to stabilize the temperature and, subsequently, an NPT ensemble for 10 ns, during which the temperature varied from 400 to 1000 K and then returned to 400 K to ensure the polymer systems attained complete amorphousness. A 10 ns NPT run was conducted at the desired temperature (400 K) to stabilize the density. The additional description of MD simulation setups can be found in Section 2 of Supporting Information.

**Figure 1:**
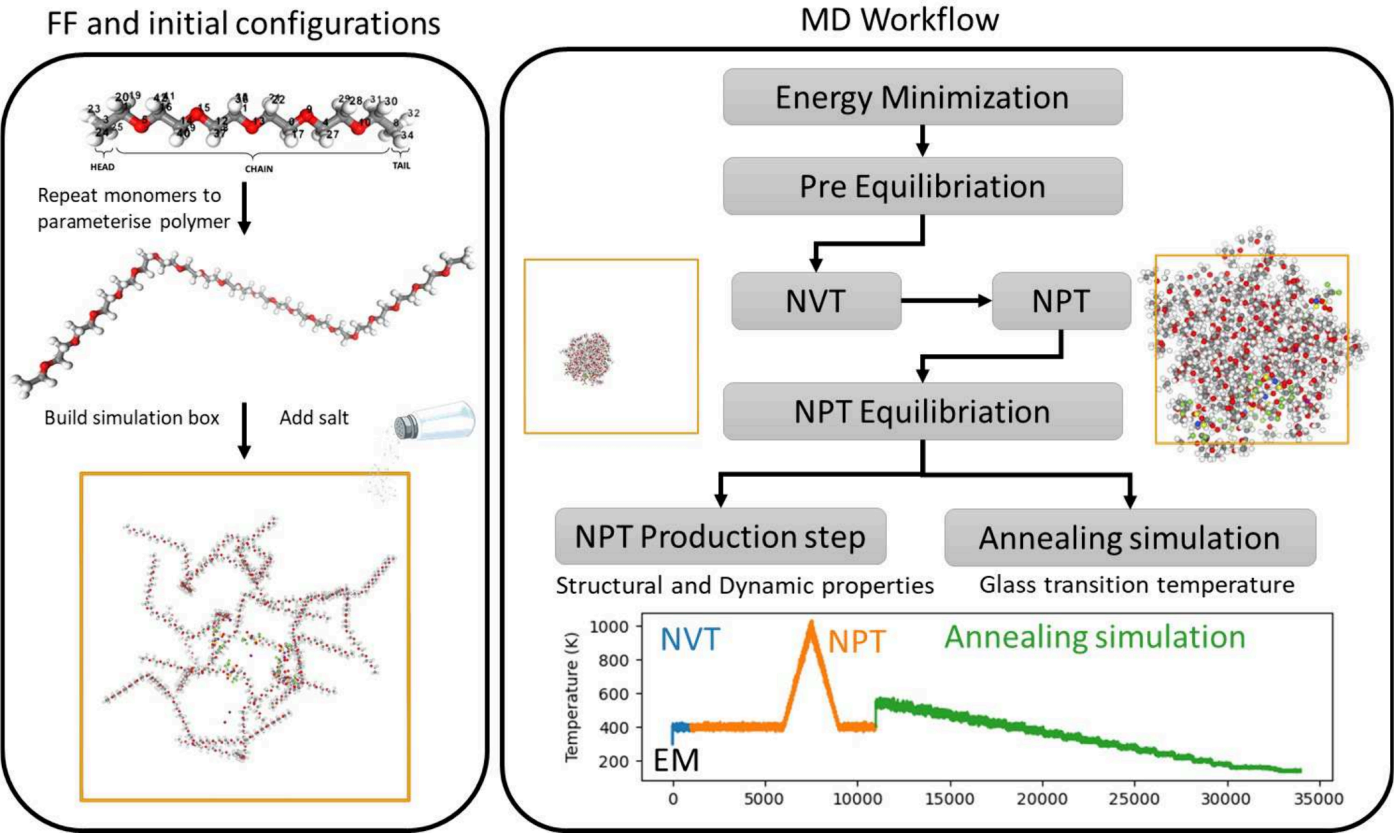
Proposed molecular dynamics simulation workflow for calculating glass transition temperature of polymer electrolytes.

From the MD simulations, *T*_g_ can be determined by monitoring the polymer electrolyte densities as a function of temperature. This transition from a soft/rubbery polymer state in the high-temperature range to a glassy/rigid state in the low-temperature range manifests as a slope change in the simulated density and temperature. The point of intersection between the fitted straight lines corresponding to the low-temperature (40–140) and high-temperature (300–440) ranges can be considered as the *T*_g_ of the simulated systems.

The first annealing simulation method involves stepwise cooling, where NPT simulations are conducted from 540 to 40 K with a step size of 20 K. At each temperature, a 2 ns equilibration followed by a 2 ns production run is performed, corresponding to a total simulation time of 100 ns. The second method employs continuous cooling simulations from 540 to 40 K at different simulation times: 0.1, 1, 10, and 100 ns, corresponding to various cooling rates of 5000, 500, 50, and 5 K/ns. To assess the impact of cooling rates, each initial cooling simulation (cool1) is followed by heating (heat1) and a second cooling simulation (cool2).

Density vs temperature profiles at different cooling rates and annealing simulations are presented in Figure 2a–d. At the highest cooling rate (5000 K/ns), significant deviations in densities are observed at higher temperatures during the heating simulation compared to the cooling simulations. This discrepancy may arise from the short simulation time, during which the polymer chains were not adequately equilibrated. As the cooling rates decrease, the observed deviation diminishes, suggesting that slower cooling rates or longer simulation times produce more accurate density profiles. Figure 2e illustrates that density profiles and *T*_g_ values from continuous cooling simulations at the lowest cooling rates (50 and 5 K/ns) closely match those from stepwise cooling simulations. The calculated glass transition temperatures differ by approximately 30 K between the various cooling rates.

**Figure 2:**
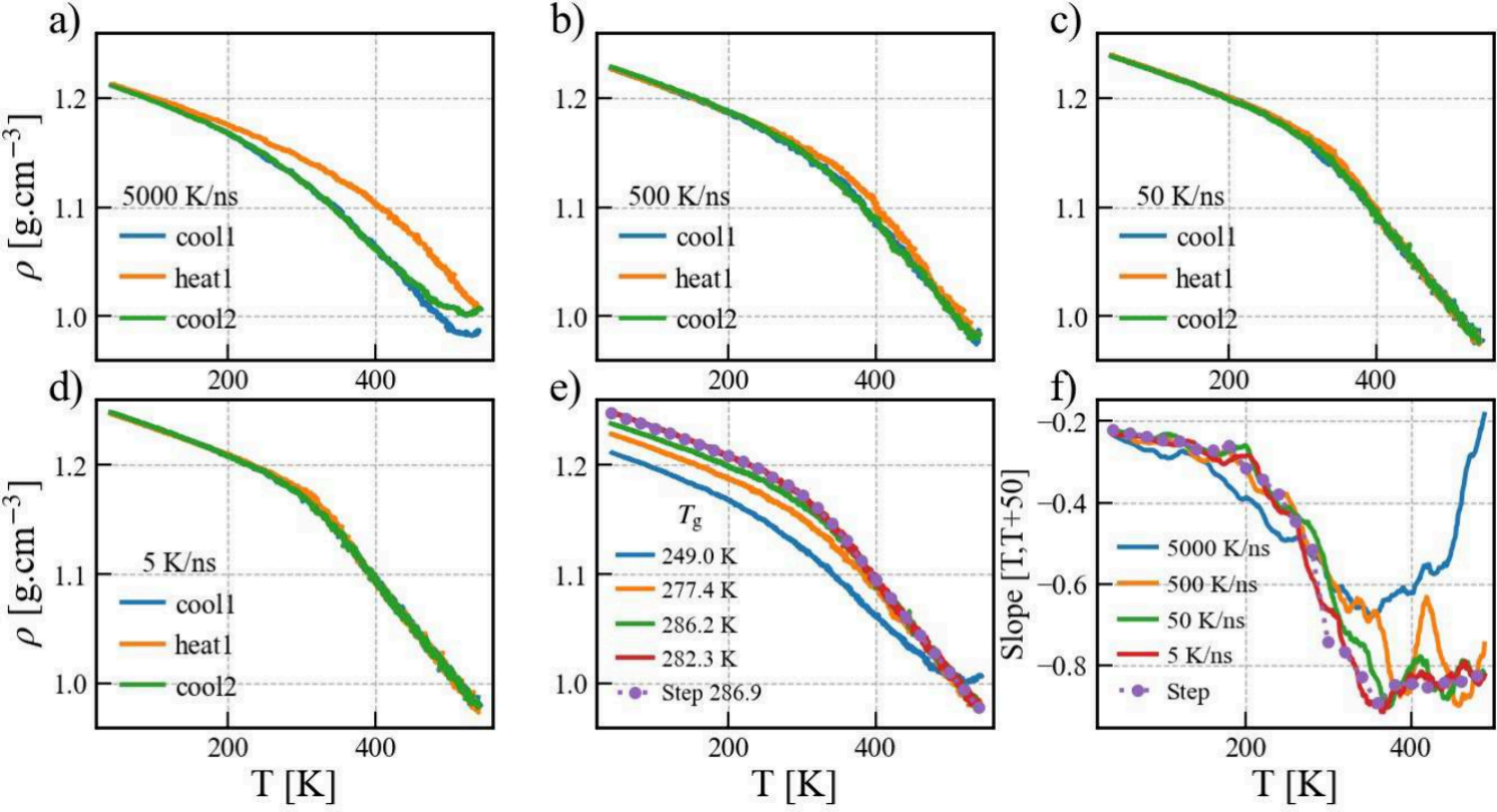
Density–temperature profiles for initial cooling (cool1), followed by heating (heat1) and final cooling annealing simulations at different cooling rates 5000 (a), 500 (b), 50 (c), 5 K/ns (d). Density profiles (e) and slopes (f) for the final cooling simulations at different rates compared with the stepwise cooling simulation.

The conventional bilinear fitting method often entails ambiguity in selecting fitting ranges for low and high temperatures, leading to potential human bias. To circumvent this issue, a slope–temperature analysis can be employed, where each point on the plot corresponds to the slope derived from a linear regression of densities within the temperature range of [*T*, *T*+50].^18^ In Figure 2f, the transitions of slopes from low to high temperatures are clearly observable for the lower cooling rate in the continuous cooling method and the stepwise cooling method, where two annealing methods give almost identical results.

To sum up, normalized temperatures relative to *T*_g_ facilitates the understanding of ion transport properties in MD simulations and enables meaningful comparisons with experimental data. Therefore, it is advisible to calculate and explicitly report *T*_g_ in computational studies of polymer electrolytes, adjusting simulated temperatures accordingly. The choice of annealing methods does impact the determination of *T*_g_, with stepwise and continuous cooling simulations yielding distinct profiles. Nevertheless, consistent results can be obtained in practice between continuous cooling method and the stepwise cooling method when a lower cooling rate and the slope-temperature analysis were used.

## Supporting Information Available

Tutorial-style descriptions of polymer Electrolyte builder for MD simulations and the corresponding MD simulation setups (PDF)
