# Supplementary material for: How to Determine Glass Transition Temperature of Polymer Electrolytes from Molecular Dynamics Simulations
Source: J Phys Chem B. 2024 Oct 21;128(43):10537–40. doi: 10.1021/acs.jpcb.4c06018 (PMC11533182; doi:10.1021/acs.jpcb.4c06018)
Supplement: Supplementary file 1 — jp4c06018_si_001.pdf [file jp4c06018_si_001.pdf]

Supporting Information: How to Determine Glass Transition Temperature of Polymer Electrolytes from Molecular Dynamics Simulations

Harish Gudla* and Chao Zhang

Department of Chemistry-Ångström Laboratory, Uppsala University, Lägerhyddsvägen 1, BOX 538, 75121 Uppsala, Sweden

E-mail: harish.gudla@kemi.uu.se

This supporting information provides necessary simulation/software details, code-snippets and visualization on building polymer electrolyte systems and setting up Molecular Dynamics simulation for glass transition temperature (T_{g}) calculation using Generalized Amber Force Field (GAFF) parameters.[1]

A more detailed step-by-step tutorials, simulation workflows, and required force field and input files for reproducing the results presented in this work can be found in this github repository GroPoB, https://github.com/Teoroo-CMC/GroPoB.

1 Polymer Electrolyte builder for MD simulations

This section provides step-by-step information on how to build initial configurations and force field parameters required for the Classical Molecular dynamics simulation using GROMACS software[2] of polymer electrolyte system PEO-LiTFSI. This tutorial is based on the Jupyter notebooks, written using different open-source tools. Before building the desired polymer electrolyte system, one has to answer the following questions:

Questions	In this work
What is the monomer?	-COCOCOCOC-
No. of monomers in the polymer chain?	25
What is the end group?	-CH_3
What is the salt?	LiTFSI
No. of polymers and salt ions	100,20

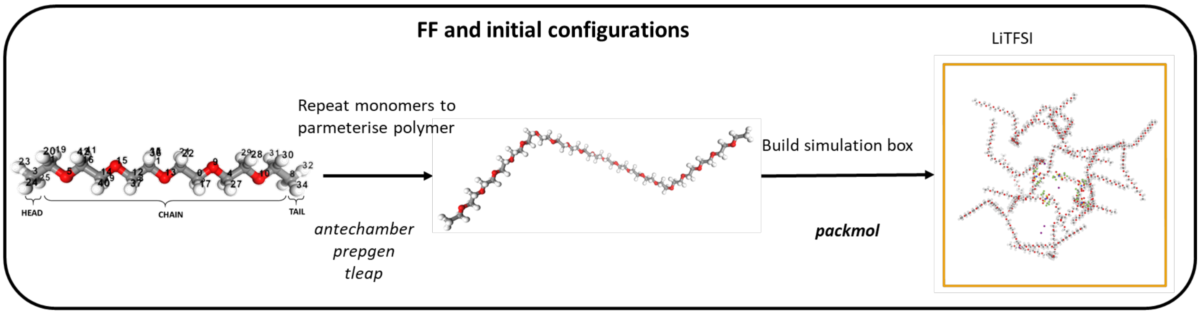

Figure S1: Scheme for building and parametrizing polymer from repeating unit.

1.1 Prerequisites

The user is assumed to be familiar with Python (Jupyter notebook) and MD simulations using GROMACS. The prerequisites for running the Jupyter notebook are:

- **GROMACS**[2]
- *ambertools*[3]
  - antechamber
  - prepgen
  - tleap
- **Python packages**
  - jupyter
  - ASE
  - nglview
- PACKMOL[4], *intermol*[5]

1.2 Step-1: Parameterizing monomer

The monomer or short polymer chain can be read from any structural file formats such as *.pdb, *.xyz, and *.gro and then ”antechamber” can be used to parameterize the monomer.

```
#Use short polymer with repeat group, head and tail group
#repeat group: COCCOCCOCCOCCOC
#head and tail group: C
#Use antechamber (ambertools) to optimize and prameterise with AM1-BCC
    charges
#antechamber options help:
#Usage: antechamber -i      input file name
#                   -fi     input file format
#                   -o      output file name
#                   -fo     output file format
#                   -c      charge method
#                   -nc     net molecular charge (int)
#                   -rn     residue name

!antechamber -i PEO_initial.pdb -fi pdb -o PEO.ac -fo ac -at gaff -an y
     -c bcc -nc 0 -rn PEO
```

The monomer/repeating unit can also be visualized using *nglview*, and atom indexes are labeled, which is helpful to define the *HEAD*,*CHAIN*, and *TAIL* parts of the monomer.

```
#Visualize the molecule
mol=read('PEO_initial.pdb')
vi=ngl.show_ase(mol);vi.add_label(radius=2,color='black',label_type='
    atomindex')
vi
```

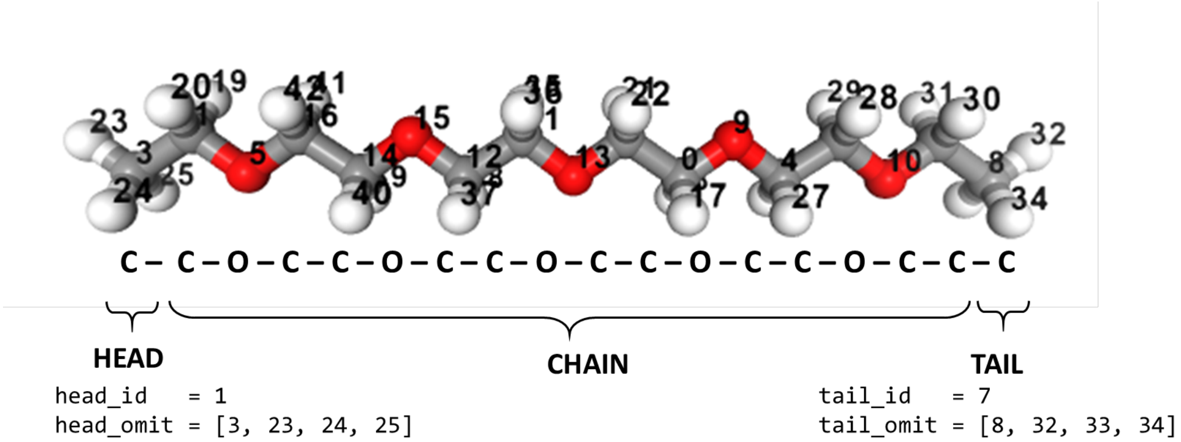

Figure S2: Repeating unit with atom index numbers, with the index numbers to define for *HEAD*, *CHAIN*, and *TAIL* parts.

1.3 Step-2: Defining the HEAD, CHAIN, and TAIL of the monomer

This is the most complicated step of this tutorial as it required to choose the two different atom indexes:

1. Head/Tail atom index numbers
2. The atom indexes needed to be omitted near Head/Tail ids.

```
#Defining CHAIN HEAD and TAIL in the monomer directory
ac=open('PEO.ac',mode='r') #Reading .ac file
[next(ac) for _ in range(2)] #Skipping first two lines of text
l=ac.readlines() #Reading lines

#Breaking the parts of monomer to CHAIN, HEAD and TAIL
#Atom index where head and tail of monomer; Check from above ngl view
    of mol
#Change these values accordingly
head_id=1
tail_id=7

head_omit=[3, 23, 24, 25] #Atoms to omit near head
tail_omit=[8, 32, 33, 34] #Atoms to omit near tail
```

Using "prepgen" we will generate three *.prepi files

1. PEO.prepi : CHAIN
2. HPT.prepi : HEAD part
3. TPT.prepi : TAIL part

Using these files, we can build the polymer chain.

1.4 Step-3: Build and parameterize single polymer chain

Here we need to set the variable “n_mono_repeat” which saves number of monomer units in the short polymer chain, and need to choose the variable “n_mono_pol” i.e., number of monomers in the desired polymer. Next, “tleap” is used to prepare the desired single polymer chain.

```
#Visualize the molecule
mol=read('PEO_initial.pdb')
vi=ngl.show_ase(mol);vi.add_label(radius=2,color='black',label_type='atomindex')
vi
```

This will generate *.prmtop and *.inpcrd files, these are coordinates and parameter files for AMBER. These files can be converted from AMBER to GROMACS using “intermol”.

```
#Build the desired homopolymer using above prepi files
%mkdir polymer
n_mono_repeat=5
n_mono_pol=25

repeat=" ".join(['PEO'] * int(int(n_mono_pol/n_mono_repeat)-2))
print('HPT '+str(repeat)+' TPT')

#Write the tleap input file to combine the preparatory files and build polymer chain
tleap=open('polymer/PEO_tleap.in','w+')
tleap.write('''source leaprc.gaff
loadamberprep PEO.prepi
loadamberprep HPT.prepi
```

```
loadamberprep TPT.prepi
mol = sequence {HPT '''+str(repeat)+''' TPT}
savepdb mol polymer/PEO_'''+str(n_mono_pol)+'''mer.pdb
saveamberparm mol polymer/PEO_'''+str(n_mono_pol)+'''mer.prmtop polymer
    /PEO_'''+str(n_mono_pol)+'''mer.inpcrd
quit''')
tleap.close()

!tleap -s -f polymer/PEO_tleap.in > polymer/PEO_tleap.out
```

Finally, visualize the polymer using nglview.

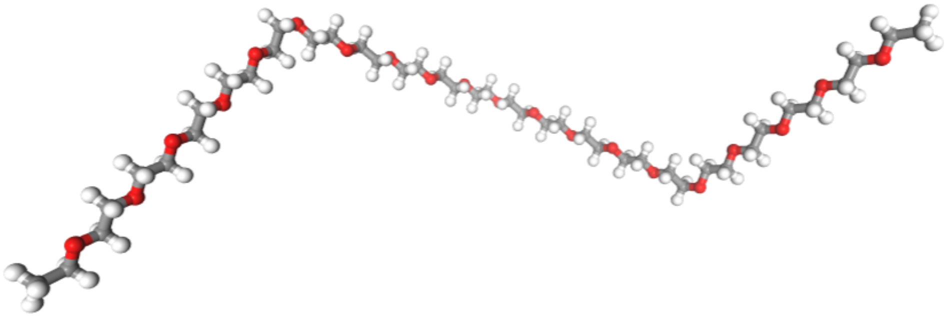

Figure S3: Visualizing the built polymer chain.

This will convert the AMBER files to GROMACS files,

1. *.gro : Initial structure file for single PEO polymer
2. *.top : Topology files consisting force field parameters.

The topology file can then be broken into two ”itp” files,

1. *ff.itp* : Consits the default settings like combinaton rules, atomtypes and LJ parameters.
2. *PEO_25mer.itp* : Consists the atomic paramerters like molecular weight, partial charges, bonded and non-bonded parameters.

1.5 Step-4: Build polymer electrolyte system

Follow the *Step-1* for desired slat ions to genertate GAFF parameters, in this turtorial we use LiTFSI, corresponding *.pdb and *.itp files were provided. We also use scaled the

partial charges on LiTFSI by a factor of 0.75.

We can now generate the topology file for PEO_LiTFSI system with desired number of polymer chains and salt concentration.

```
### Make the topology file with ITP directory location and system details
ITPDIR='/home/harish/GroPolBul/tutorial/ITP'
npol=40;nmon=25;conc=0.08
nions=npol*nmon*conc
topol=open('topol.top','w+')
topol.write('''#include "'''+str(ITPDIR)+'''/ff.itp"
#include "'''+str(ITPDIR)+'''/ITP/PEO_'''+str(nmon)+'''mer.itp"
#include "'''+str(ITPDIR)+'''/ITP/li_75c.itp"
#include "'''+str(ITPDIR)+'''/ITP/tfsi_75c.itp"

[ system ]
PEO_LiTFSI_'''+str(conc)+'''

[ molecules ]
polymer   '''+str(npol)+'''
LI     '''+str(nions)+'''
TFS    '''+str(nions)+'''
''')
topol.close()
```

By using PACKMOL, we can now generate the initial configuration file "output.pdb" of the MD simulation box.

```
#Build initial simulation boxes using packmol
pack=open('packmol.inp','w+')
pack.write('''tolerance 2.0
filetype pdb
output initial.pdb
structure PEO_25mer.pdb
  number '''+str(npol)+'''
  inside cube 0. 0. 0. 100.
end structure
```

```

structure li.pdb
  number '''+str(nions)+'''
  inside cube 20. 20. 20. 50.
end structure

structure tfsi.pdb
  number '''+str(nions)+'''
  inside cube 20. 20. 20. 50.
end structure
        ''')
pack.close()
!packmol < packmol.inp
```

2 MD simulation setups

The initial configuration file ("initial.pdb") and topology file ("topol.top") generated in the above section can be used to perform the MD simulations, and the required input files (*.mdp files) can be found in the GitHub page. The schematics of the MD protocol is shown in Fig. 1 of Main text and the used MD settings are detailed here. During the energy minimization step, the maximum force to stop the minimization is set to 10 nmkJ/mol. The Bussi–Donadio–Parrinello thermostat[6] and a Berendsen barostat[7] were used for NVT and NPT ensembles, where the coupling constants for both the thermostat and the barostat were set to 1 ps. The long-range electrostatic interactions were employed through a particle mesh Ewald technique[8] with an interpolation order of 4 and a grid size of 0.4 nm. The short-range cutoff distances of the van der Waals and Coulombic interaction in the direct space are 1.2 nm. The bonds involving hydrogen atoms were constrained using the LINCS algorithm[9]. For all MD simulations, the leapfrog integrator was employed with a time step of 1 fs.

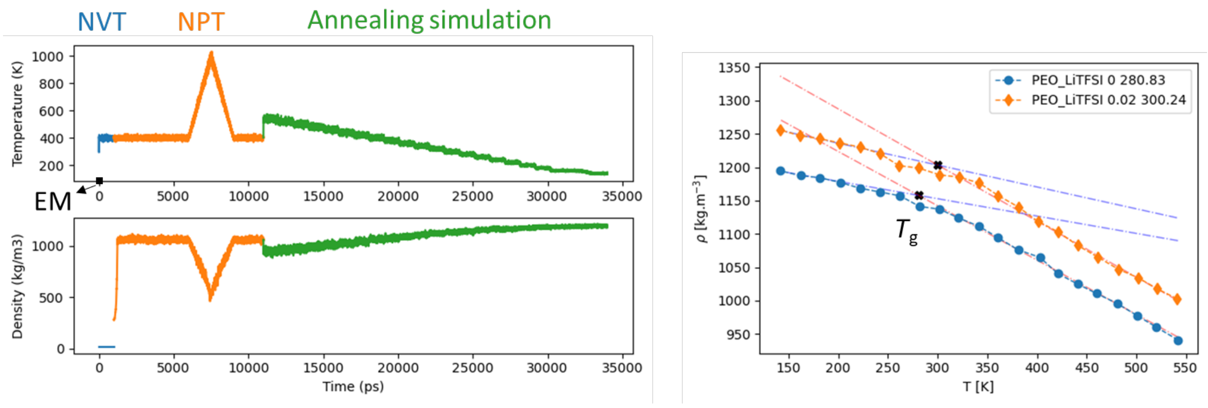

Figure S4: (left) The density (kg/m^3) and temperature (K) as function of simulation time (ps) during energy minimization (EM), NVT and NPT equilibrations and step-wise annealing simulation. (right) The density (ρ) vs. temperature (T) plots from step-wise annealing simulations used to calculate the glass transition temperatures (T_g) for PEO-LiTFSI systems.

References

1 Wang, J.; Wolf, R. M.; Caldwell, J. W.; Kollman, P. A.; Case, D. A. Development and testing of a general amber force field. *Journal of Computational Chemistry* **2004**, *25*, 1157–1174.

2 Abraham, M. J.; Murtola, T.; Schulz, R.; Páll, S.; Smith, J. C.; Hess, B.; Lindahl, E. GROMACS: High performance molecular simulations through multi-level parallelism from laptops to supercomputers. *SoftwareX* **2015**, *1-2*, 19–25.

3 Case, D. A.; Aktulga, H. M.; Belfon, K.; Cerutti, D. S.; Cisneros, G. A.; Cruzeiro, V. W. D.; Forouzesh, N.; Giese, T. J.; Götz, A. W.; Gohlke, H.; et al. AmberTools. *Journal of Chemical Information and Modeling* **2023**, *63*, 6183–6191.

4 Martínez, L.; Andrade, R.; Birgin, E. G.; Martínez, J. M. PACKMOL: A package for building initial configurations for molecular dynamics simulations. *Journal of Computational Chemistry* **2009**, *30*, 2157–2164.

5 Shirts, M. R.; Klein, C.; Swails, J. M.; Yin, J.; Gilson, M. K.; Mobley, D. L.; Case, D. A.; Zhong, E. D. Lessons learned from comparing molecular dynamics engines on the SAMPL5 dataset. *Journal of Computer-Aided Molecular Design* **2016**, *31*, 147–161.

6 Bussi, G.; Donadio, D.; Parrinello, M. Canonical sampling through velocity rescaling. *The Journal of Chemical Physics* **2007**, *126*, 014101.

7 Berendsen, C.; Postma, M.; Gunsteren, v.; DiNola, A.; Haak, J. R. Molecular dynamics with coupling to an external bath. *The Journal of Chemical Physics* **1984**, *81*, 3684–3690.

8 Darden, T.; York, D.; Pedersen, L. Particle mesh Ewald: An Nlog(N) method for Ewald sums in large systems. *The Journal of Chemical Physics* **1993**, *98*, 10089–10092.

9 Hess, B.; Bekker, H.; Herman; Johannes LINCS: A linear constraint solver for molecular simulations. *Journal of Computational Chemistry* **1997**, *18*, 1463–1472.
